# Docking and Molecular Dynamics Study to Identify Novel Phytobiologics from *Dracaena trifasciata* against Metabolic Reprogramming in Rheumatoid Arthritis

**DOI:** 10.3390/life12081148

**Published:** 2022-07-29

**Authors:** Shanzay Ahmed, Peter John, Rehan Zafar Paracha, Attya Bhatti, Monica Guma

**Affiliations:** 1 Department of Healthcare Biotechnology, Atta-ur-Rahman School of Applied Biosciences (ASAB), National University of Sciences and Technology (NUST), Islamabad 44000, Pakistan; sahmed.phdabs16asab@asab.nust.edu.pk (S.A.); attyabhatti@asab.nust.edu.pk (A.B.); 2 School of Interdisciplinary Engineering & Sciences (SINES), National University of Sciences and Technology (NUST), Islamabad 44000, Pakistan; rehan@sines.nust.edu.pk; 3 Department of Medicine, Division of Rheumatology, Allergy and Immunology, University of California San Diego, 9500 Gilman Drive, San Diego, CA 92093, USA; mguma@health.ucsd.edu

**Keywords:** *Dracaena trifasciata*, GLS1, HK2, metabolic reprogramming, rheumatoid arthritis

## Abstract

Enhancement of glycolysis and glutaminolysis are the two most common modalities associated with metabolic reprogramming in rheumatoid arthritis (RA). This enhancement is concomitant to the upregulation of hexokinase 2 (HK2) and glutaminase 1 (GLS1). Hence, the current study was undertaken to identify potential phytobiological inhibitors against HK2 and GLS1, from *Dracaena (Sansevieria) trifasciata*, an indigenous ethnomedicinal plant found in Pakistan, using computational analysis. Phytobiologics from *Dracaena trifasciata* were assessed for their ability to co-inhibit HK2 and GLS1 via molecular docking and molecular dynamics simulations. The results underscored seven phytobiologics with promising binding affinities for both HK2 and GLS1. Molecular dynamics simulations further elucidated that all seven identified phytobiologics inhibited HK2 by forming stable complexes but only five amongst the seven had the potential to form stable complexes with GLS1 in real time, thereby implying the potential of co-inhibition for these five compounds. Compound 28MS exhibited an equally strong binding profile for both HK2 (−8.19 kcal/mol) and GLS1 (−8.99 kcal/mol). Furthermore, it exhibited a similar trend in stability during simulation for both targets. Our results serve as a primer for a more lucid understanding towards co-inhibition of HK2 and GLS1 using multiple computational approaches. The identified phytobiologics should undergo *in-vitro* and *in-vivo* validation to corroborate their therapeutic potential in RA.

## 1. Introduction

Rheumatoid arthritis (RA) is an autoimmune disorder that affects approximately 1% of the population worldwide [1]. The onset of the disease is best described by the formation of the pannus, entailing the hyperplastic synovium ensuing bone and cartilage erosion [2]. RASFs (rheumatoid arthritis synovial fibroblasts) exhibit varying characteristics in terms of gene expression and morphology in contrast to normal fibroblasts. Nutrient competition coupled with the stress concomitant in the inflamed joint incite metabolic reprogramming in the underlying fibroblasts to safeguard their survival [3,4,5].

Metabolic reprogramming is a complex biological response that is regulated by an augmented enzyme expression. It sustains the physiological and pathological changes that inhabit the RA synovium. RASFs mediate the bone and cartilage destruction in rheumatoid arthritis. The phenotypic metamorphosis from inactive to metabolically aggressive fibroblasts such as synoviocytes (FLS), necessitate an escalated biosynthetic requisite. This is concurrent with deviations in cellular metabolism and bioenergetic nexus that provide for accelerated proliferation and fuels increased migration, invasion, and pro-inflammatory mediator production in the RA joint [6,7]. Proliferating cells shift from oxidative phosphorylation to aerobic glycolysis and glutaminolysis, thus greatly enhancing the breakdown of both glucose and glutamine [8,9]. Augmented levels of glycolysis enable the cells to meet their escalated energy demand. Glutaminolysis, on the other hand, provides the necessary biosynthetic precursors to keep up with the increased metabolism. These enhancements are concurrent with elevated expressions of hexokinase 2 (HK2) and glutaminase 1 (GLS1) in RA.

Hexokinase 2 has a high, but inducible expression in adipose, skeletal, and cardiac tissues. Inhibiting HK2 mitigated inflammation and reduced cartilage obliteration in the K/BxN model of arthritis. Elevated expression of HK2 in RAFLS delivers a migratory and invasive leverage that is eradicated with the extirpation of HK2 [10]. Currently, HK2 inhibitors are unavailable; however, it can be successfully inhibited with 2 deoxy glucose (2DG) and 3 Bromopyruvate (3-BrPA) and its derivatives [6,11]. Augmented glycolysis shifts the metabolism towards production of lactate from pyruvate rather than its entry into the TCA cycle. To recompense, RAFLS relies on elevated glutaminolysis [6]. Torres, et al. [12] and Takahashi, et al. [13] reported that obstructing GLS1, the enzyme responsible for the conversion of glutamine to glutamate, directly checks RAFLS proliferation, thereby mitigating the pathological intensity of the autoimmune disease, arthritis. Escalated levels of glutamate populate the synovial fluid of patients affected with RA. This directly associates with a raised level of IL-6 [14]. GLS1 inhibitor, CB-839 is currently being assessed for its efficacy in clinical trials against prostate cancer. Hence, inhibition of these metabolic enzymes provides cell-specific, novel biological therapeutic targets for the development of novel compounds against RA.

The existing medications against RA, on more than one occasion, have produced undesirable fallouts [15]. Moreover, the existing biological therapy against the disorder bears financial restraints. Additionally, a good number of patients fail to respond efficiently [16,17]. Thus, the therapeutic potential must outweigh even the minutest of the side effect to be deemed as successful. Conventional therapies that clear the preliminary scrutiny and are permitted for human treatment do not assure total relief from RA. Therefore, rigorous research to develop safe and effective drugs for RA is the need of the hour. Several plants have been reported to be effective against RA [18,19]. Plants provide a plethora of promising phytochemicals for targeted therapies. *Dracaena (Sansevieria) trifasciata* is an important ethnomedicinal plant that is readily available at a very economical price in Pakistan. Literature associates anti-diabetic, anti-tumor, anti-oxidant, and anti-microbial activities with species of this genus thus corroborating its pharmacological potential [20]. However, elucidation of the functional role and cellular mechanisms by which compounds from *Dracaena trifasciata* may modulate the metabolic reprogramming in RA remains untapped in the literature.

Individual inhibition of HK2 and GLS1 has been successfully attempted with numerous different inhibitors. Nonetheless, there are currently no phytobiological inhibitors that target both HK2 and GLS1. Dual inhibition would probably imply a greater remission in the symptoms of the disease in contrast to inhibiting a single target. Computational approaches show assurance in the optimization of drug development thus transforming clinical research. Hence, the current study was undertaken to identify a set of novel yet highly potent phytobiological inhibitor leads from *Dracaena trifasciata* against the underlying metabolic reprogramming in RA by targeting HK2 and GLS1 via computer aided drug designing (CADD) (Figure S1). Specifically, we aimed to investigate the amino acids that would most dominantly interact with effective HK2 and GLS1 inhibitors. Targets based on specific interactions would pave the way towards safer and more directed therapeutics.

## 2. Materials and Methods

### 2.1. Collection and Identification

*Dracaena (Sansevieria) trifasciata* was selected based upon its previously reported ethnomedicinal significance [21,22,23]. *Dracaena trifasciata* was obtained from a plant nursery in Lahore. The plant was verified by taxonomists from NUST and QAU, Islamabad, Pakistan. Moreover, the herbarium of the entire plant was prepared with inflorescence and submitted to Pakistan Museum of National History (PMNH) Islamabad under Voucher No 00046224.

### 2.2. Extract Preparation

Leaves of *Dracaena trifasciata* were obtained and air dried after removing the extraneous material. Dried leaves were mechanically powdered and macerated with ethanol and methanol (Sigma Aldrich, St. Louis, MO, USA), respectively, in 1:10 for four weeks in dark. The macerated mixtures were then filtered by employing Whatmann^®^ (Maidstone, UK), grade 41 filter paper followed by rotary evaporation to concentrate them. The rotary evaporated extracts were further air dried in dark to eliminate any traces of moisture. Dried ethanolic (DT_E_) and methanolic extracts (DT_M_) were stored at 4 °C in dark bottles for future use.

### 2.3. Biochemical Testing of Extracts

Phytochemical analysis of DT_E_ and DT_M_ was performed by following the methods explained by [24,25,26,27]. Oxidative stress, one of the salient features of RA, is responsible for the underlying metabolic reprogramming. Hence, the anti-oxidant activity of extracts was assessed by employing Diphenyl-1-picrylhydrazyl (DPPH) assay by adapting the method of Andhare, Raut, and Naik [21].

### 2.4. Gas Chromatography–Mass Spectrometry Analysis

Gas chromatography–mass spectroscopy (GC–MS-QP2010 Ultra) with DB-5 MS chromate graphic column (Diameter 0.25 mm, thickness 0.25 mm and Length 30 mm) was used to evaluate the composition of DT_E_ and DT_M_. A total of 1 μL of samples was injected by employing the injection port maintained at 200 °C in a split ratio of 5:1. Helium, with a flow rate of 1 mL/min, was employed as a carrier gas. For 1 min, the column temperature was held at 40 °C and then programmed at 5 °C per minute to 280 °C and then held for 5 min. The MS-ion source was kept at a temperature of 200 °C, 70 eV electron energy, and the interface temperature of GC–MS was maintained at 200 °C with a scan range of 35–800 *m*/*z*. Identification of the volatiles was made by paralleling their relative abundances and mass spectra with NIST 11.

### 2.5. Drug like Properties

With the advancement of CADD, few methods have gained significant prominence as they successfully predict the potential of the compound (ligand) as an orally active drug [28]. Hence, compounds obtained via GC–MS were subjected to Lipinski [29], Ghose [30], Veber [31], Egan [32], Muegge [33], and Egan’s BOILED Egg [34] to underscore their favorability in biological systems by employing web-based accessible tool SwissADME [35] (http://www.swissadme.ch/index.php, accessed on 27 June 2022). Moreover, pharmacokinetics (Gastrointestinal Absorption, Blood Brain Barrier Permeance and Cytochrome P450 inhibition) was also calculated by SwissADME. AdmetSAR another web-based accessible tool [36,37] (http://lmmd.ecust.edu.cn/admetsar2/, accessed on 27 June 2022) was used to establish a toxicity profile of the compounds.

### 2.6. Structure Retrieval

Three-dimensional structures of compounds satisfying the drug likeliness were retrieved from PubChem Database [38], whereas those unavailable on PubChem were drawn on ChemDraw Ultra 12 [39]. Three-dimensional structures for controls of HK2 and GLS1 inhibitors, 3-BrPA, 2DG, and CB839, respectively, were also downloaded from PubChem. All the structures were then converted into PDB format by using Open Babel, version 2.3.1 [40].

### 2.7. Preparation of Targets

X-ray diffraction crystal structures of both targets HK2 (2NZT) and GLS1 (3VP1) with resolution of 2.45 Å and 2.30 Å, respectively, were obtained from RCSB Protein Databank [41,42,43] (https://www.rcsb.org/, accessed on 27 June 2022). Prior to docking, Discovery Studio 2016, was employed to prepare both the targets for docking by removing native ligands and water molecules. Additionally, Auto dock tools (ADT) [44] was utilized to commute polar hydrogens and Kollman charges in order to augment the susceptibility for electronegative atoms.

### 2.8. Active Site Prediction

Prior to docking, active site prediction of HK2 and GLS1 was carried out by DOGsite scorer (dogsite.zbh.uni-hamburg.de, accessed on 27 June 2022), which explores the physiochemical and geometric properties of the binding pockets and estimates the associated drug-like properties [45,46].

### 2.9. Docking and Protein Ligand Interaction Profiling

Docking facilitates the screening of compounds by predicting target-ligand interactions at a molecular level without prior knowledge of the existing modulators for the target. Docking was performed for potential drug-like compounds with HK2 and GLS1. Auto Dock 4.2, a suite of automated docking tools, was employed to generate 50 different binding conformations per compound (ligand). All the rotatable bonds of the ligands under study were randomized prior to docking and consequently treated as flexible during the runs of the docking whereas both targets were kept rigid. Lamarckian genetic algorithm [47] was used to perform docking studies. Grid box to locate the ligand was set at x = 0.47, y = −20.33, z = 26.05 for target 1 (2NZT:HK2); and x = 7.90, y = −4.48, and z = −11.85 for target 2 (3VP1:GLS1). Grid spacing was set at 0.375 Å, whereas the dimensions of the grid were set to 60 Å × 60 Å × 60 Å. Low binding energies indicate a high ligand–protein interaction and vice versa. Hence, the top seven ligands that showed binding energies higher than the controls (3-BrPA and 2 DG for HK2 and CB-839 for GLS1) against both targets were selected for pose analysis.

### 2.10. Pose Analysis

Based on binding energy, the best pose for the seven inhibitors exhibiting promising activity against both HK2 and GLS1 was selected for protein–ligand interaction profiling (PLIP). Protein–ligand interactions were performed by the web-based Protein Ligand Interaction Profiler [45,48] (https://projects.biotec.tu-dresden.de/plip-web/plip, accessed on 27 June 2022) to analyze the possible types of non-covalent interactions.

### 2.11. MD Simulations

Molecular dynamics simulations were performed using GROMACS 5.1 [49,50], a command-based software, to elucidate dynamic behavior coupled with binding mode and confirmational stability of the obtained hits against both targets. Docking complexes selected for both targets were subsequently converted to gro file to preserve default topology compliant with default force field. CHARMM forcefield was selected [51]. The protein was restricted in a rhombic dodecahedron cubic box to facilitate accommodation of solvent molecules. The protein was then positioned in the center of the cube having a 1nm distance from the boundary of the cube ensuing a periodic image 2 nm apart. The solvent used for simulation was water spc216.gro, having a force constant (k_pr_) 1000 kJ mol^−1^ nm^−2^. Genion [52] was employed to remove charges on the protein by the addition of counter ions. The energy minimization (n steps = 5000) was carried out using the steepest descent approach for each protein–ligand complex. Electrostatic force was applied and the cut-off scheme used was Verlet. Moreover, the energy-minimized structure was obtained at the lowest coordinate point. NVT and NPT ensembles for temperature and pressure equilibrations were carried out for 100 ps. The equilibrated and stabilized complexes were then subjected to molecular dynamics simulations for 30 ns.

The trajectory analysis was performed using GROMACS (Lindahl et al., 2001). Root mean square deviation (RMSD) was taken as a measure to assess the structural stability of each complex. The root-mean-square fluctuation (RMSF) of the α-carbon was used to monitor the structural flexibility and movement. Moreover, radius of gyration was employed as a measure of the conformational changes (compactness), by examining how unfolded or folded the target under study was. Lastly, hydrogen bond analysis was performed to commute the stability of favorable interactions. The number of hydrogen bonds with respect to their occupancies directly relate to the binding strength of the phytobiologic with its target. Hydrogen bond analysis was performed using VMD 1.9.3 (Theoretical and Computational Biophysics Group, University of Illinois, Urbana-Champaign) [53] the cut-off distance was set at 4.1 Å and the angle cut-off between donor and H-bond acceptor atoms was set to ± 120°. Residues constituting the top ten percentage occupancies of H-bonds were considered as stable contacts between the target and the inhibitor.

## 3. Results

### 3.1. Phytobiological Screening and Radical Scavenging Activity of the Extracts

**Figure 1:**
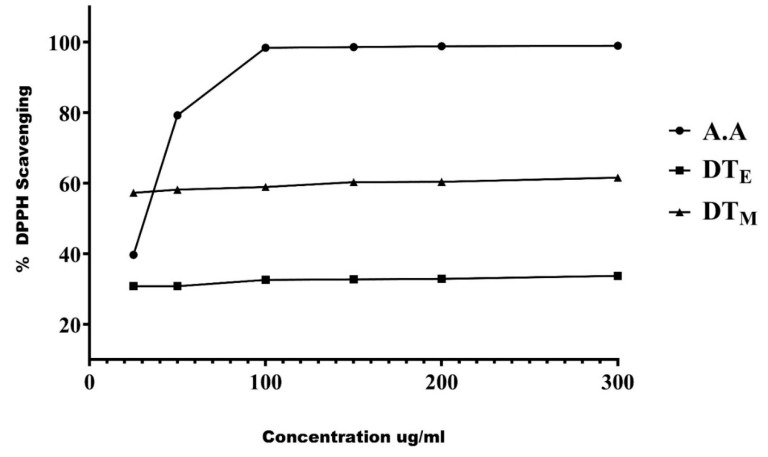
**Free radical scavenging activity of the extracts of *Dracaena trifasciata*.** The free radical scavenging activity of ethanolic (DT_E_) and methanolic extracts (DT_M_) of *Dracaena trifasciata* augmented with increasing concentration of the extract thereby underscoring its potential antioxidant effect. Linear regression was performed to analyze the results. The data was obtained from three independent observations and has been presented as mean.

Phytobiologics have immense therapeutic significance; therefore, the prepared ethanolic and methanolic extracts of the plant were screened for their presence. Both the extracts had a plethora of medicinally important phytobiologics, as shown in Table 1. Alkaloids, flavonoids, tannins, glycosides, steroids, sterols, and triterpenes were common to both the extracts, whereas coumarins and glycosides were exclusive to methanolic extract (DT_M_) and leucoanthocyanins, and phenols and diterpenes were unique to the ethanolic extract (DT_E_). Six different concentrations of both the extracts were tested to assess the radical scavenging of the extracts. An increase in scavenging activity was observed with increasing concentrations of the extracts. The extracts, however, were less active in scavenging free radicals compared to ascorbic acid a highly antioxidant compound used as a standard in this experiment (Figure 1).

### 3.2. Library Generation and Drug Likeness Analysis

A library of potential leads (phytobiologics) with drug-like properties was established by screening the obtained compounds from GC–MS analysis against the parameters of drug likeness. The compounds that passed the drug likeliness filters, were then proceeded for molecular docking analysis with HK2 and GLS1 as their biological activity of interest against our desired targets in RA remains unestablished (Supplementary File S1).

### 3.3. Molecular Docking

In silico molecular docking against HK2 and GLS1, crucial to metabolic reprogramming in RA was achieved by Autodock 4.2 (The Scripps Research Institute, La Jolla, CA, USA). The results indicated the presence of promising inhibitors inside the pockets of HK2 and of GLS1. Successful inhibitors formed varying numbers of hydrophobic interactions, hydrogen bonds, salt bridges, and pi cation interactions with the targets (Supplementary File S2). The inhibitors were than ranked based on binding energies. Ideally, a promising inhibitor should have a lower value of binding affinity, thereby establishing a strong interaction on the specified site with the target. Results indicated the presence of seven inhibitors displaying the most robust binding energies against both targets in our library (Table 2 and Supplementary File S3). The binding affinities of these inhibitors surpassed the binding affinities of the used controls 3-BP (−3.10 kcal/mol), 2 DG (−3.21 kcal/mol), and CB-839 (−7.62 kcal/mol).

**Figure 2:**
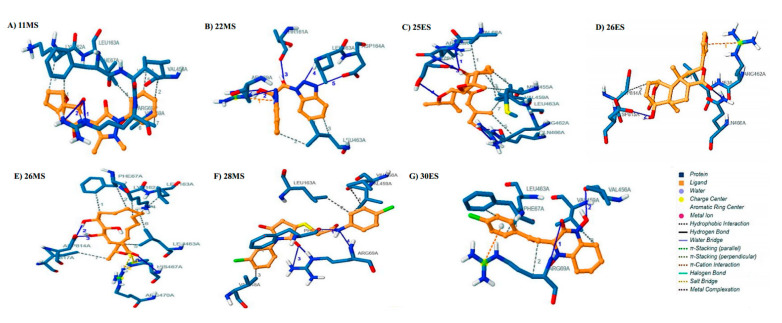
**Docked poses and molecular interactions of the proposed inhibitors with HK2.** Interaction analysis of inhibitors (brown) at the binding site of HK2 (blue) visualized by protein ligand interaction profiler: (**A**) 11MS, (**B**) 22MS, (**C**) 25ES, (**D**) 26ES, (**E**) 26MS, (**F**) 28MS, and (**G**) 30ES.

Phytobiologic 11MS constituted a binding energy of −8.34 kcal/mol because of two hydrogen bonds and seven hydrophobic interactions. The side chain of Arg69 was responsible for the formation of two hydrogen bonds(2.92 Å, 3.97 Å). The amino acids Phe67, Val68, Lys162, Leu163, Val456, and Val459 engaged in hydrophobic interactions with 11MS(Figure 2A). The binding energy of 22MS was calculated to be −7.06 kcal/mol for HK2. Side chains of Arg69 (3.11 Å,3.12 Å) and Asp164 (3.68 Å) formed a total of three hydrogen bonds with 22MS. Additionally, main chains of Phe67 (3.39 Å), Thr161 (2.96 Å), and Leu163 (2.85 Å) formed three hydrogen bonds each with the phytobiologic. Hydrophobic interaction was observed for amino acid Leu463. Arg69 also engaged in pi cation interaction with 22MS (Figure 2B). 25ES was docked to HK2 with a binding energy of −7.85 kcal/mol. A single hydrogen bond was constituted by a main chain of Arg69 (2.82 Å) whereas Ser70 formed three hydrogen bonds via its main and side chain, respectively (2.88 Å, 3.87 Å, and 2.87 Å). Gln466 (3.16 Å, 3.04 Å) and Arg462 (3.66 Å) side chains were also responsible for the formation of three hydrogen bonds. Amino acids involved in hydrophobic interactions were Val68, Met455, Val459, Arg462, and Leu463 (Figure 2C). 26ES showed a binding affinity of −7.41 kcal/mol for HK2. The exhibited binding energy was a result of hydrogen bonds and hydrophobic and cation–π interactions. The hydrogen bonds were formed by side chains of Arg462 (3.01 Å, 3.67 Å), Gln466 (3.99 Å), and Asp815 (2.87 Å), respectively. Leu463 and Asp814 interacted via hydrophobic interaction with 26ES. The presence of aromatic ring in the structure of 26ES also allowed the phytobiologic to interact with Arg462 of HK2 via cation–π interaction (Figure 2D). Phytobiologic 26MS was bound to HK2 with a binding energy of −7.05 kcal/mol. Three hydrogen bonds, seven hydrophobic interactions, and two salt bridges surfaced because of this interaction. 26MS formed a hydrogen bond with main chain of His467 (3.98 Å). Two hydrogen bonds were formed by side chain interactions of Asp 814 (2.97 Å, 2.54 Å). Phe67, Lys162, Leu163, Leu463, and Ile817 were dominantly involved in hydrophobic interaction with 26MS. Moreover, His467 and Arg470 were also critically involved in salt bridge formation with the phytochemical 26MS (Figure 2E). 28MS yielded a binding energy of −8.19 kcal/mol with HK2. This interaction surfaced four hydrogen bonds in total. The phytobiologic formed two hydrogen bonds with side chains of Arg69 (2.80Å,3.23 Å) and one hydrogen bond each with main chains of Phe67 (2.61 Å) and Arg69 (3.41 Å). Furthermore, Phe67, Leu163, Val248, Val456, and Val 459 of the target protein formed hydrophobic interactions with 28MS (Figure 2F). The overlapping presence of Phe67 signifies its prime position in the interaction between 28MS and HK2. 30ES was bound with the HK2 enzyme with a binding energy of −7.86 kcal/mol. Side chains of Arg69 (3.99 Å) and Val456 (3.28 Å) each formed two hydrogen bonds with 30ES. Furthermore, hydrophobic interactions were constituted by Phe67, Arg69, Val459, and Leu463. A cation–π interaction was also observed with Arg69 for 30ES. (Figure 2G).

**Figure 3:**
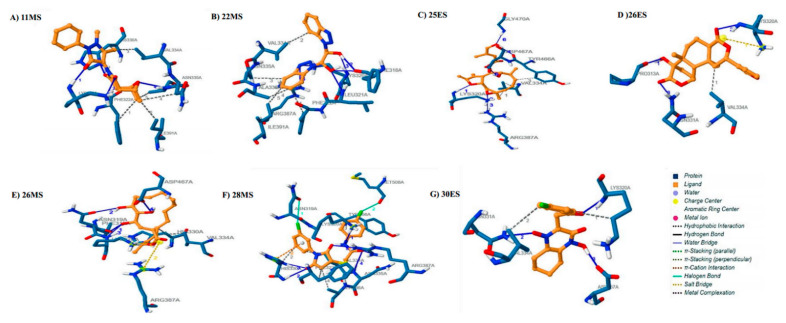
**Docked poses and molecular interactions of the proposed inhibitors with GLS1.** Interaction analysis of inhibitors (brown) at the binding site of GLS1 (blue) visualized by protein ligand interaction profiler (**A**) 11MS, (**B**) 22MS, (**C**) 25ES, (**D**) 26ES, (**E**) 26MS, (**F**) 28MS, and (**G**) 30ES.

The interaction of 11MS with GLS1 yielded four hydrogen bonds and seven hydrophobic interactions which constituted a binding energy of −7.81 kcal/mol for the target. Lys320 (2.86 Å, 3.22 Å) interacted via its main and side chain to form two hydrogen bonds. Side chain of His330 (2.52 Å) and main chain Ala336 (3.44 Å) each formed one hydrogen bond with 11MS. Phe322, His330, Val334, Asn335, Ala336, and Ile391 all were actively engaged in hydrophobic interactions with 11MS (Figure 3A). The phytochemical interacted in a likewise manner with GLS1 constituting a binding energy of 7.79 kcal/mol. Phe318 (2.79 Å, 3.49 Å), Lys320 (2.95 Å), and Leu331 (3.04) interacted through their main chains to form a total of four hydrogen bonds with 22MS. Interaction of side chains Lys320 (3.17 Å) and Arg387 (3.37 Å) further surfaced two hydrogen bonds. Additionally, Phe322, Val334, Asn335, Ala336, Ile391 were involved in interacting with 22MS through hydrophobic interactions (Figure 3B). Moreover, six hydrogen bonds and three hydrophobic interactions were responsible for a binding energy of −9.04 of 25ES with GLS1. The side and main chain of Lys320 formed a total of two hydrogen bonds (3.04 Å, 2.74 Å) with the oxygen atoms of 25ES. Two hydrogen bonds resulted as a consequence of interaction of the side chains of Arg387 (3.88 Å) and Asp467 (3.04 Å) with 25ES. Furthermore, the remaining two hydrogen bonds were a result of interaction of Tyr466 (3.16 Å) and of Gly470 (3.04 Å) with 25ES. The amino acids, Val334 and Tyr466, were critically involved in hydrophobic interactions with 25ES (Figure 3C). 26ES was bound to GLS1 with a binding energy of −8.75 kcal/mol. Side chains of Asn331 (3.07 Å) and Arg387 (2.76 Å, 3.62 Å) formed a total of three hydrogen bonds. Main chain interactions of Pro313 (2.79 Å) and Lys320 (3.06 Å) also resulted in the formation of hydrogen bonds. Hydrophobic interaction was established between Val334 and 26ES. Apart from hydrogen bonding, Lys320 further formed a salt bridge with 26ES (Figure 3D). Four hydrogen bonds, two hydrophobic interactions, and salt bridges cumulatively exhibited a binding energy of −8.01 kcal/mol for 26MS. Three hydrogen bonds were formed with successive amino acids, namely Phe318 (3.22 Å), Asn319 (3.17 Å), and Lys320 (2.70 Å) in the binding pocket of GLS1 whereas the fourth one was formed with the side chain Asp467 (2.76 Å). Amino acids involved in hydrophobic interactions were His330 and Val334 whereas the amino acids Lys320 and Arg387 interacted with 26MS to form salt bridges (Figure 3E). Hydrogen bonds, hydrophobic interactions alongside halogen bonds, and π -cation interactions were observed when 28MS was docked to GLS1. Lys320 (3.83 Å), His330 (2.84 Å), and Arg 387 (3.33 Å, 2.97 Å interacted with the phytobiologic through their side chains to form hydrogen bonds. Additionally, the phytobiologic also formed hydrogen bonds with the main chain of Asn335 (3.99 Å) and His330 (3.52 Å). Hydrophobic interactions with Phe322, His330, Val334, Ala336, and Tyr466 were also observed. Other than hydrogen bonds, Lys320 and His330 also interacted with the aromatic rings of 28MS via cation–π interaction. Halogen bonds were also existent due to the interaction of oxygen atoms of Asn319 and Met508 with Cl of 28MS. All these contributed ominously towards a binding affinity of −8.99 kcal/mol (Figure 3F). The binding energy of 30ES with GLS1 was calculated to be 9.47 kcal/mol. Hydrogen bonds were observed between 30ES and the main chain of Lys320 (2.75 Å) and side chains of Asn331 (2.89 Å) and Asp467 (2.95 Å). Val 334 and Lys320 participated in hydrophobic interactions with 30ES (Figure 3G).

### 3.4. Molecular Dynamics Simulations

The best binding conformation of the seven phytobiologics with the least binding energy in the complex with HK2 and GLS1 were subjected to molecular dynamics simulations to elucidate binding strength, structural stability of the complex, and non-covalent interaction patterns.

**Figure 4:**
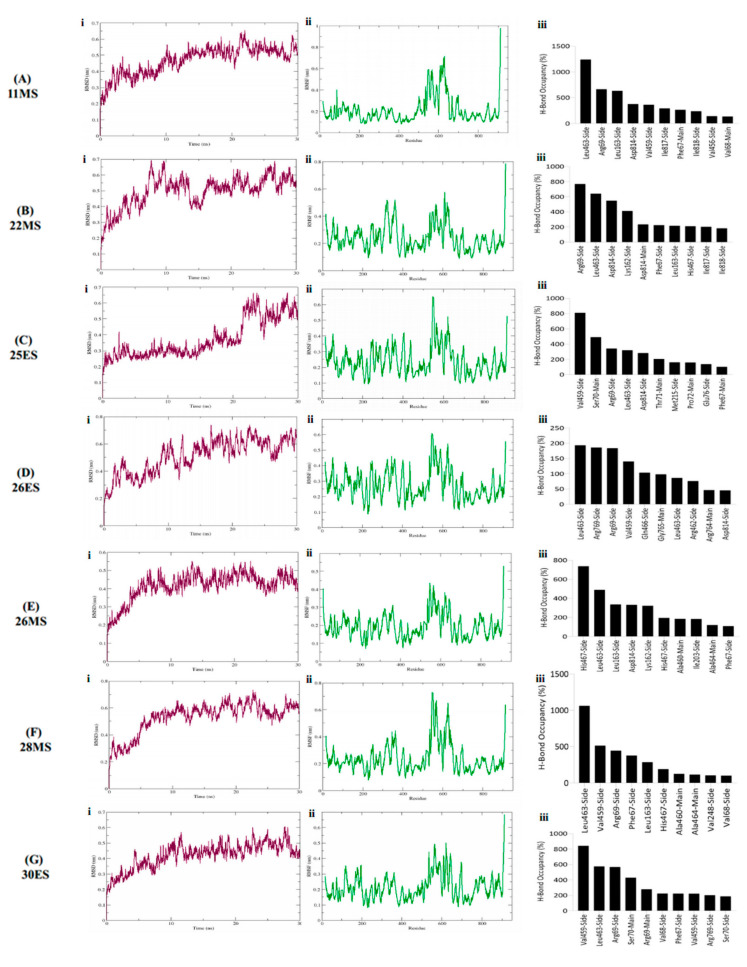
RMSD plot (complex), RMSF plot (backbone) and residues wise percentage of H-Bond occupancy for entire simulation span of HK2 in the complex with (**A**) 11MS, (**B**) 22MS, (**C**) 25ES, (**D**) 26ES, (**E**) 26MS, (**F**) 28MS and (**G**) 30ES.

All the simulated inhibitors in the complex with HK2 exhibited an average RMSD range between 0.5 nm to 0.65 nm (Figure 4Ai–Gi). Slight fluctuations were observed between 5–10 ns, and, then 25–30 ns but since they were in the permissible limit of variation, 0.1–0.3 nm, hence, were considered non-significant. Rings, twists, coils, and loops in a protein are prone to fluctuations. To pinpoint these areas of flexibility, fluctuation of each residue was calculated for the entire duration of the simulation. There was no sign of flexibility in the active site of the protein (Figure 4Aii–Gii). Minor fluctuations were observed throughout the protein whereas the major fluctuations were observed in the loop and areas other than the active site. The amino acids associated with major fluctuations were Leu, Glu, Lys, Gly, Ala and Thr positioned from a range of 359–364 and 530 to 630. Variations observed in R_g_ were in a range from 3.9–4.2 nm conveying a general trend of protein stability during simulation (Figure S2).

Molecular dynamics simulations further elucidated that not all the hydrogen bonds identified during docking for HK2 persisted. New bonds were formed which were then sustained in the trajectory. Relatively strong hydrogen bonding was detected for 28MS and 11MS with HK2 in contrast to other phytobiologics. The hydrogen bond formed by Arg69 initially during docking persisted during simulation for both 11MS and 22MS. Additionally, Leu463, Leu163, and Asp814 formed the highest occupancy, ant the most stable H-bonds with these inhibitors. Other notable amino acids forming hydrogen bonds with notable occupancies were Asp814, Val459, Ile817, Ile818, Phe67, Leu162, and His467 (Figure 4Aiii,Biii). Unlike 11MS and 22MS, the hydrogen bond constituted by Arg69, was detected at a much lower occupancy for 25ES. Ser70 formed the only other preserved hydrogen bond for 25ES. New yet stable hydrogen bonds for 25ES were observed with Val459, Leu463, Asp814, Thr71, Met215, Pro72, and Phe67 (Figure 4Ciii). Furthermore, assessment revealed 26ES to be the inhibitor with an overall lowest hydrogen bond occupancy in contrast to all other inhibitors. Hydrogen bonds formed by Arg462 and Gln466 were overlapping between docking and simulations for 26ES. However, they were not considered stable because of their exceedingly low occupancies (Figure 4Diii). Hydrogen bonds formed between inhibitor 26MS and amino acids His467 and Asp814 were common to both docking and simulation. Moreover, like 11MS and 22MS, 26MS also established stable hydrogen bonds with Leu163 and Leu463 (Figure 4Eiii). For inhibitor 28MS, the most significant hydrogen bonding occurred with amino acids Phe67, Arg69, Leu463, Val459 and His467. Out of these, the hydrogen bonds formed by Phe67 and Arg69 were preserved from docking. 30ES also exhibited a hydrogen bond pattern similar to 28MS in terms of amino acids involved in the formation of stable interactions (Figure 4Fiii,Giii).

**Figure 5:**
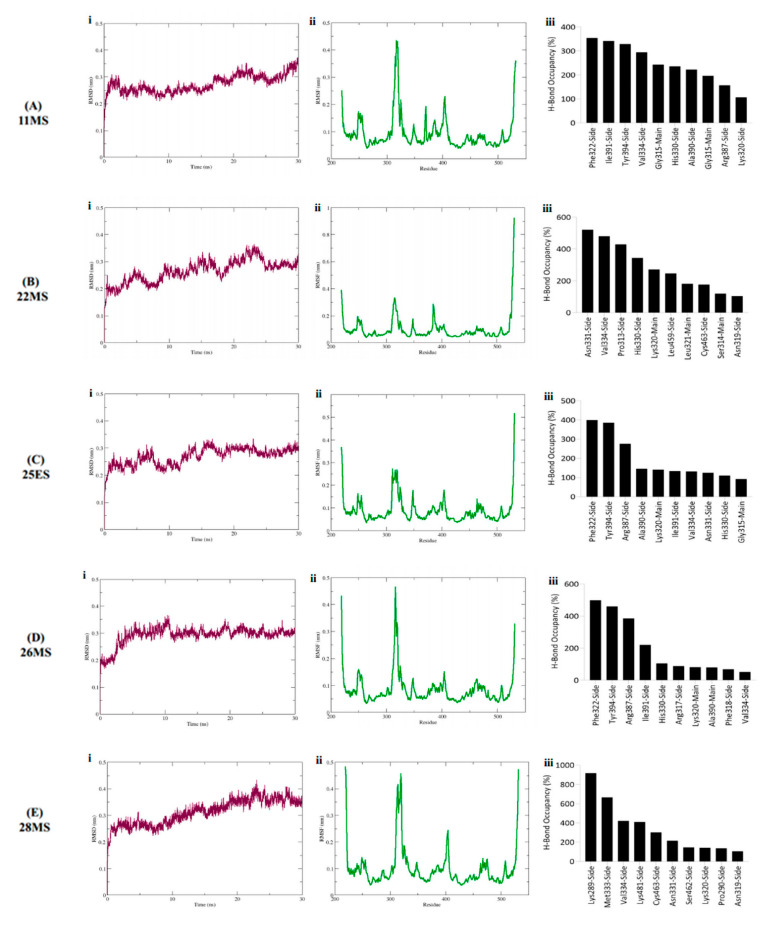
RMSD plot (complex), RMSF plot (backbone) and residues wise percentage of H-Bond occupancy for entire simulation span of GLS1 in the complex with (**A**) 11MS, (**B**) 22MS, (**C**) 25ES, (**D**) 26MS, and (**E**) 28MS.

In case of GLS1, both 26ES and 30ES displayed erratic behavior in their RMSDs thus limiting their efficacy as promising inhibitors for GLS1. As opposed to this, ligands 11MS, 22MS, 25ES, 26MS and 28MS exhibited maximum stability RMSD within a range of 0.31 nm to 0.35 nm when in the complex with GLS1 (Figure 5Ai–Ei). No significant fluctuations were observed in the RMSD of these five ligands. Furthermore, most of these complexes converged to an equilibration state in the last 5 ns of the simulation Additionally, all inhibitors under study with GLS1 exhibited a similar trend in the RMSF with slight fluctuation being owed to the type of inhibitory compound in the complex with the protein. Overall, for all systems, the loop regions displayed the highest values of RMSF. Maximum RMSF values were obtained for amino acid Lys positioned from 314–317 and 400–415 (Figure 5Aii–Eii). R_g_ in the range of 1.9–2.0 nm underscored stability of the protein during the simulation span (Figure S3).

Similar to HK2, both new and old bonds were detected for all the phytobiological inhibitors against GLS1. The hydrogen bond analysis of GLS1 inhibitors demonstrates that 28MS and 22MS formed the greatest number of stable bonds with the highest occupancy as opposed to the others. For inhibitor 11MS, the only hydrogen bond that was retained from docking with a relatively lower occupancy was formed by Lys320. All other detected bonds were new and were formed during the simulation. Phe322, Ile391, and Tyr394 formed the most stable high occupancy H-bonds for 11MS (Figure 5Aiii). The hydrogen bond formed by Lys 320 was also preserved for 22MS in addition with Leu321. Other stable high occupancy bonds for 22MS were owed to amino acids Asn331, Val334, Pro313, and His330 (Figure 5Biii). For 25ES and 26MS, hydrogen bonds formed by Lys320, and Phe318 were the only ones found to be common to both docking and simulation. However, the retention of the hydrogen bond detected with Arg387 was observed only for 25ES but not 26MS. New bonds for both these inhibitors were found with amino acids Phe322 and Tyr394 (Figure 5Ciii,Diii). The phytobiologic inhibitor 28MS formed the highest occupancy stable hydrogen bonds with GLS1, suggesting maximum inhibition of the target by this inhibitor. All the hydrogen bonds detected for this inhibitor except for Lys320 were newly established. The occupancy and stability of these new bonds was highest in contrast to all other inhibitors under investigation. The bonds executed by Lys289 and Met333 were the most notable ones for 28MS (Figure 5Eiii).

## 4. Discussion

RA is a chronic systemic autoimmune disease described by synovitis of the affected joints [54]. Even with the best management, patients affected by RA may experience progression of the disorder, thus, requiring hip replacement surgery with a total hip implant. This bears an economic burden which further worsens the scenario for patients affected with RA [55,56]. The immune microenvironment in RA instigates metabolic reprogramming of FLS, thus switching them from a static to a rather metabolically active state to meet the energy demands. Activated FLS exhibit an invasive, migratory, and aggressive phenotype, thus causing joint and cartilage destruction and bone erosion. These metabolic changes are thus promising therapeutic targets for RA [3,57]. HK2 and GLS1 sit at the heart of metabolic reprogramming as elevated glucose and glutamine metabolism synergistically makes provision for the aggressive behavior of the RAFLS. The existing side effects of current medications compel the choice to search for new and safer therapies without limiting their efficacy Recently, phytochemicals have been employed in the treatment of RA. The literature reports *D. Tracifcatica* to be ethnomedicinally important but its therapeutic efficacy in RA, remains unexplored. Thus, the current study was undertaken to elucidate the effect of novel phytobiologics from *D. Tracifcatica* against HK2 and GLS1 in RA.

Inflammatory cells inhabiting the synovial joint develop an inflammatory milieu in the extracellular matrix that induces cellular infiltration and generation of reactive oxygen species (ROS), and consequently, leading to the development of oxidative stress and state of hypoxia [58,59]. Additionally, the free radicals induce a metabolic shift in RASFs, thus disturbing the homeostasis leading to the damage of the basic articular structures [60]. Phytochemical analysis revealed the *D. Trifastica* possess as a profusion of phytochemicals which are known for their antioxidant effects. DPPH assay further corroborated this by establishing both ethanolic (DT_E_) and methanolic extracts (DT_M_) as powerful antioxidants. Therefore, in this study we rationalized that DT_E_ and DT_M_ may possess valuable phytobiological inhibitors to treat RASF’s metabolism: RA’s Achilles heel.

Docking and simulations have proved to be a substantial tool in the process of drug discovery. In our case, docking assisted in shortlisting the potential phytobiologics that had the co-inhibition potential for HK2 and GLS1 from our data set. The simulations on the other hand further validated their role by elucidating an understanding of interaction in terms of bonding and stability with respect to time. To validate our molecular docking analysis, docking was also performed using known inhibitors of both the targets, 3-BP and 2DG for HK2 and CB839 for GLS1. All the controls exhibited weaker binding energies than the proposed inhibitors, thus further strengthening their therapeutic efficacy. MD simulations largely elucidate the confirmational stability, a phenomenon that largely influence the effective inhibition of target proteins by therapeutic compounds. Owing to their erratic RMSD while in the complex with GLS1, both 26ES and 30ES were deemed as unsuccessful for the inhibition of GLS1 in real time. However, they were still regarded as potential leads for HK2. The indicated variation in stability of the same inhibitor while in the complex with two different targets delineates the delicate intricacy of inhibiting the target of interest.

Hydrogen bonds are considered to be extremely specific interactions between the inhibitor and the target. These bonds are imperative in determining the stability of the target and inhibitor complex. They also ascertain metabolism and drug specificity. It was observed that Phe67, Val68, Arg69, Ser70, Leu163, Val 459, Leu463, Gln466, and Ile817 exhibited effective binding interactions with the phytobiological inhibitors in docking for HK2. The interactions constituted by these amino acids were considered stable as their presence was dominantly detected in the simulations as well. It could be hypothesized that these residues play a crucial part in the generation of a local environment which helps in the identification of the inhibitors from our data set [61,62]. Furthermore, earlier studies indicate the presence of an active site and allosteric site for GLS1. The binding at these pockets inhibits the catalytic activity of the enzyme. Docking revealed that our phytobiological inhibitors are attached at these very sites to achieve the desired inhibition. Residues including Tyr249, Gln285, Ser286, Asn335, Glu381, Asn388, Tyr414, Tyr466, Val484, Phe318, Lys320, Leu321, Phe322, Leu323, Asn324, Glu325, and Tyr394 have been identified as crucial for GLS1 binding through X-ray crystallographic studies and site-directed mutagenesis [63]. Likewise, our docking and simulation results are well in line with the previously reported studies that have shown the importance of residues Pro313, Phe318, Lys320, Leu321, Phe322, Val334, Asn335, Ala336, Ile391, Arg387, and Tyr 466 for interaction with the inhibitors and substrates of GLS1.

The current study is a pioneer in successfully underscoring the effect of novel phytobiologics against metabolic reprogramming. All the identified inhibitors fulfill the requisite parameters of drug likeness and pharmacokinetics, least binding energies, and stable interactions thereby acting as potential HK2 and GLS1 inhibitors. Having said that, computational techniques prove exceedingly fruitful in the search of potential leads from a large data set as ours. They are efficient and cost and time effective. Nonetheless, they do not mirror the precise biological conditions, henceforth, *in-vitro* and *in-vivo* validation mechanisms are indispensable to further cement the therapeutic potential of these phytobiologics. A metabolomic analysis by employing more reliable techniques such as NMR will prove fruitful in profiling the substrate and product levels of the target enzymes to elucidate the extent of inhibition of these compounds. The promising novel inhibitors and their predicted associations will pave the way towards improved prognosis and disease management. This will give researchers valuable insights to develop plant-derived biologically active molecules as treatment options.

## 5. Conclusions

FLS in RA rely heavily on glucose and glutamine metabolism, subsequently increasing the expression of HK2 and GLS1. Thus, these metabolic enzymes present themselves as novel therapeutic targets for RA. Herein, a set of phytobiologics from *Dracaena trifasciata* were screened by molecular docking against HK2 and GLS1 to elucidate their binding affinity for effective inhibition. The binding affinity of the phytobiologics was also compared with the controls 3-BrPA, 2-DG, and CB-839. Finally, the relative stability of seven lead phytobiologics showing greater binding affinities than used controls was validated by MD simulations. All complexes displayed structural stability for HK2, whereas only five displayed stability, for GLS1 during the 30 ns MD simulation period. Thus, the outcome of this study demonstrates that the screened phytobiologics may be potential drug candidates against metabolic reprogramming in RA by targeting key enzymes HK2 and GLS1. These phytobiologics should be further exploited *in-vitro* and *in-vivo* to develop improved therapeutics against RA, which is the need of the hour.

## Figures and Tables

**Table 1:** Phytobiologic analysis of methanolic (DT_M_) and ethanolic extracts (DT_E_) of *Dracaena trifasciata*.

		(DT_M_)	(DT_E_)
1	Alkaloids	+	+
2	Phenols	−	+
3	Anthraquinones	−	−
4	Flavonoids	+	+
5	Anthocyanins	−	−
6	Leucoanthocyanins	−	+
7	Tannins	+	+
8	Phlobatannins	−	−
9	Coumarins	+	−
10	Terpenoids	−	−
11	Diterpenes	−	+
12	Triterpenes	+	+
13	Steroids	+	+
14	Sterols	+	+
15	Saponins	−	−
16	Resins	−	−
17	Emodins	−	−
18	Glycosides	+	+
19	Cardiac glycosides	+	−

Note: (−) Not present; (+) Present.

**Table 2:** Binding energies in kcal/mol of phytobiologics from *Dracaena trifasciata* after docking with HK2 and GLS1.

Compound Name	Target 1 (HK2)		Target 2 (GLS1)	
	Binding Energy (kcal/mol)	Inhibition Constant (Ki)	Binding Energy (kcal/mol)	Inhibition Constant (Ki)
**11MS**	−8.34	776.71 nM	−7.81	1.90 μM
**22MS**	−7.06	6.64 μM	−7.79	1.94 μM
**25ES**	−7.85	1.77 μM	−9.04	237.37 nM
**26ES**	−7.47	3.33 μM	−8.75	383.40 nM
**26MS**	−7.05	6.84 μM	−8.01	1.35 μM
**28MS**	−8.19	998.63 nM	−8.99	257.91 nM
**30ES**	−7.86	1.74 μM	−9.47	113.85 nM

## Data Availability

The following information was supplied regarding data availability: raw data are available in the Supplemental files.

## Supplementary Materials

The following supporting information can be downloaded at: https://www.mdpi.com/article/10.3390/life12081148/s1, Figure S1: Workflow of the conducted study; Figure S2: Radius of gyration (R_g_) of target protein (HK2) for the entire simulation span whilst in the complex with (A) 11MS, (B) 22MS, (C) 25ES, (D) 26ES, (E) 26MS, (F) 28MS and (G) 30ES Figure S3: Radius of gyration (R_g_) of target protein (GLS1) for the entire simulation span whilst in the complex with (A) 11MS, (B) 22MS, (C) 25ES, (D) 26MS and (E) 28MS. File S1: Library of lead compounds (phytobiologics); File S2: Molecular interactions during molecular docking of the proposed inhibitors (phytobiologics) with HK2 and GLS1. File S3: Shortlisted seven phytobiologics from the library of lead compounds.

## Abbreviations

RA	Rheumatoid Arthritis
RASF	Rheumatoid Arthritis Synovial FIbroblasts
HK2	Hexokinase 2
GLS1	Glutaminase 1
2DG	2 Deoxyglucose
3-BrPA	3 Bromopyruvate
IL-6	Interleukin-6
CADD	Computer Aided Drug Designing
DT_M_	Dried Methanolic Extracts
DT_E_	Dried Ethanolic Extracts
DPPH	Diphenyl-1-picrylhydrazyl
PDB	Protein Data Bank
PLIP	Protein Ligand Interaction Profiling
MD Simulations	Molecular Dynamics Simulations
GROMACS	Groningen Machine for Chemical Simulations
CHARMM	Chemistry at Harvard Macromolecular Mechanics
