# Supplementary material for: Docking and Molecular Dynamics Study to Identify Novel Phytobiologics from Dracaena trifasciata against Metabolic Reprogramming in Rheumatoid Arthritis
Source: Life (Basel). 2022 Jul 29;12(8):1148. doi: 10.3390/life12081148 (PMC9410489; doi:10.3390/life12081148)
Supplement: Supplementary file 1 [file life-12-01148-s001.zip › Supplemental Figures S1-S3.pdf]

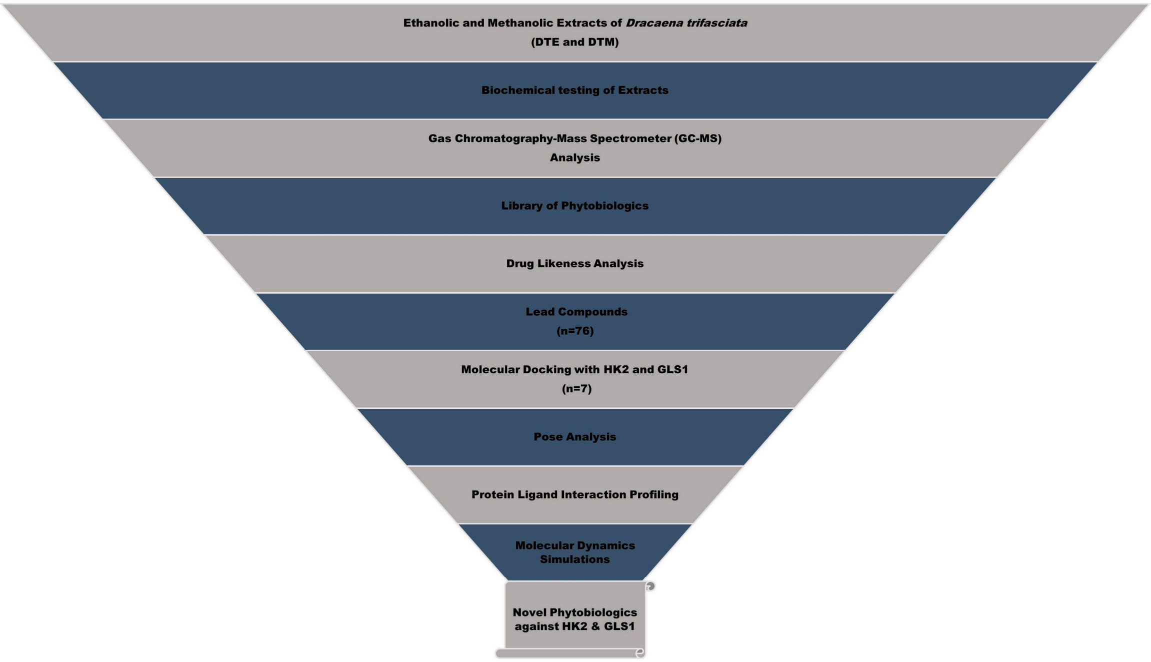

Figure S1. Workflow of the conducted study.

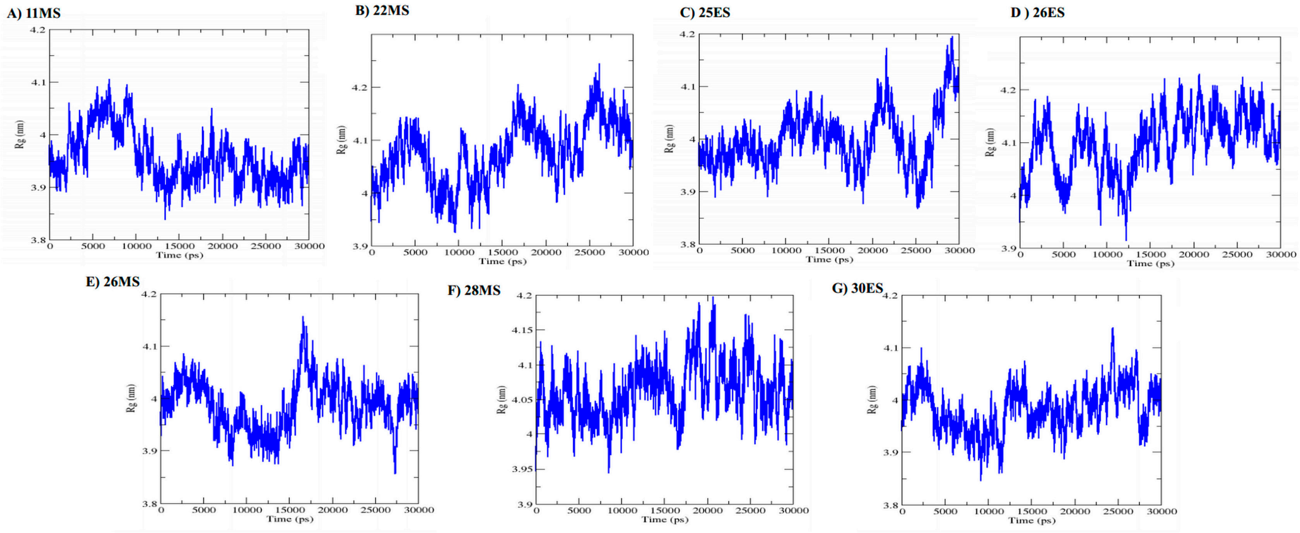

Figure S2. Radius of gyration (Rg) of target protein (HK2) for the entire simulation span whilst in the complex with (A) 11MS, (B) 22MS, (C) 25ES, (D) 26ES, (E) 26MS, (F) 28MS and (G) 30ES.

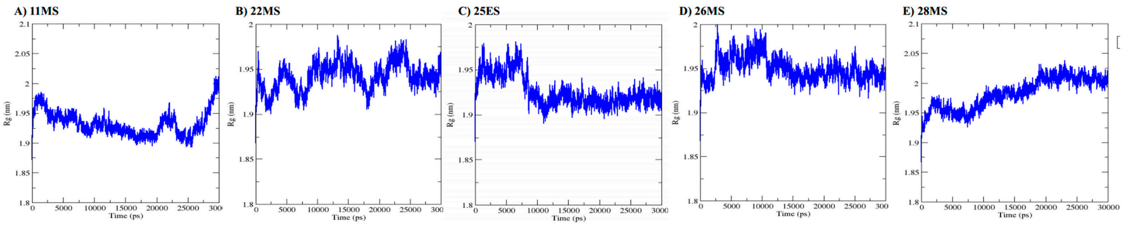

Figure S3. Radius of gyration (Rg) of target protein (GLS1) for the entire simulation span whilst in the complex with (A) 11MS, (B) 22MS, (C) 25ES, (D) 26MS and (E) 28MS
