# Supplementary material for: Docking and Molecular Dynamics Study to Identify Novel Phytobiologics from Dracaena trifasciata against Metabolic Reprogramming in Rheumatoid Arthritis
Source: Life (Basel). 2022 Jul 29;12(8):1148. doi: 10.3390/life12081148 (PMC9410489; doi:10.3390/life12081148)
Supplement: Supplementary file 1 [file life-12-01148-s001.zip › Supplemental File S2.pdf]

1. Hydrogen Bond Interactions of Lead Compounds for HK2

Compound	Residue	Amino Acid	Distance		Donor Angle	Donor atom	Acceptor atom
			H-A	D-A			
11MS	69A	ARG	1.94	2.92	160.16	521 [Ng+]	8141 [O2]
	69A	ARG	3.31	3.97	124.05	527 [Ng+]	8141 [O2]
22MS	67A	PHE	3.06	3.39	101.39	8135 [Nar]	497 [O2]
	69A	ARG	2.16	3.12	156.27	527 [Ng+]	8132 [O3]
	69A	ARG	2.13	3.11	159.97	521 [Ng+]	8135 [Nar]
	161A	THR	2.03	2.96	159.25	8144 [O3]	1334 [O2]
	163A	LEU	1.87	2.85	160.35	1352[Nam]	8147 [Nar]
	164A	ASP	3.20	3.68	113.29	1368 [O3]	8148 [Nar]
25ES	69A	ARG	1.96	2.82	139.53	513 [Nam]	8158 [O3]
	70A	SER	2.36	2.88	110.15	530 [Nam]	8158 [O3]
	70A	SER	3.21	3.87	126.02	8158 [O3]	534 [O2]
	70A	SER	2.47	2.87	105.27	536 [O3]	8144 [O2]
	462A	ARG	2.94	3.66	128.37	4159[Ng+]	8156 [O3]
	466A	GLN	2.35	3.16	135.93	4201[Nam]	8156 [O3]
	466A	GLN	2.19	3.04	144.88	8156 [O3]	4200 [O2]
26ES	462A	ARG	2.01	3.01	165.78	4165[Ng+]	8154 [O2]
	462A	ARG	2.86	3.67	136.24	4159[Ng+]	8154 [O2]
	466A	GLN	2.99	3.99	167.68	4201[Nam]	8132 [O3]
	815A	ASP	2.06	2.87	139.63	8156 [O3]	7259 [O2]
26MS	467A	HIS	3.36	3.98	120.51	4204[Nam]	8150 [O2]
	814A	ASP	2.03	2.97	160.44	8153[O3]	7250[O2]
	814A	ASP	1.72	2.54	137.58	8155[O3]	7250[O2]
28MS	67A	PHE	2.11	2.61	108.39	8142[Nam]	497[O2]
	69A	ARG	2.15	2.80	119.45	521 [Ng+]	8131[O2]
	69A	ARG	2.69	3.23	113.10	527 [Ng+]	8131[O2]
	69A	ARG	2.64	3.41	131.61	513 [Nam]	8142 [Nam]
30ES	69A	ARG	3.22	3.99	133.15	513[Nam]	8141[O2]
	456A	VAL	2.58	3.28	128.17	8153 [O3]	4104 [O2]

2. Hydrogen Bond Interactions of Lead Compounds for GLS1

Compound	Residue	Amino Acid	Distance (Å)		Donor Angle	Donor atom	Acceptor atom
			H-A	D-A			
11MS	320A	LYS	1.84	2.86	173.23	963 [Nam]	2925 [O2]
	320A	LYS	2.25	3.22	157.68	972 [N3+]	2944 [O2]
	330A	HIS	2.06	2.52	104.87	1072 [Npl]	2935 [O2]
	336A	ALA	2.45	3.44	163.25	1124[Nam]	2941 [O3]
22MS	318A	PHE	2.01	2.79	135.13	2938 [O3]	944 [O2]
	318A	PHE	2.51	3.49	173.51	2926 [O3]	944 [O2]
	320A	LYS	1.97	2.95	162.19	963 [Nam]	2926 [O3]
	320A	LYS	2.15	3.17	174.90	972 [N3+]	2942 [Nar]
	321A	LEU	2.52	3.04	111.03	976 [Nam]	2938 [O3]
	387A	ARG	2.48	3.37	145.69	1618[Ng+]	2942 [Nar]
25ES	320A	LYS	2.03	3.04	170.07	963[Nam]	2938[O2]
	320A	LYS	1.80	2.74	152.27	972 [N3+]	2936[O3]
	387A	ARG	3.14	3.88	130.45	1618[Ng+]	2936[O3]
	466A	TYR	2.19	3.16	172.80	2950[O3]	2331[O2]
	467A	ASP	2.11	3.04	158.80	2952[O3]	2348[O2]
	470A	GLY	2.17	3.04	142.40	2370[Nam]	2950[O3]
26ES	313A	PRO	1.98	2.79	139.69	2950 [O3]	897 [O2]
	320A	LYS	2.14	3.06	148.70	963 [Nam]	2926[O3]
	331A	ASN	2.11	3.07	155.15	1086[Nam]	2925 [O3]
	387A	ARG	1.75	2.76	169.20	1618[Ng+]	2948 [O2]
	387A	ARG	2.93	3.62	125.58	1615[Ng+]	2948 [O2]
26MS	318A	PHE	2.40	3.22	140.07	2949 [O3]	944 [O2]
	319A	ASN	2.39	3.17	137.00	2947 [O3]	959 [O2]
	320A	LYS	1.79	2.70	147.53	963 [Nam]	2949 [O3]
	467A	ASP	1.97	2.76	135.60	2945 [O3]	2348 [O2]
28MS	320A	LYS	2.89	3.83	154.68	972 [N3+]	2936 [Nam]
	330A	HIS	2.56	3.52	156.69	1065[Nam]	2926 [O2]
	330A	HIS	1.86	2.84	159.38	1072 [Npl]	2926 [O2]
	335A	ASN	3.03	3.99	156.51	1113[Nam]	2935 [O2]
	387A	ARG	2.51	3.33	137.45	1618[Ng+]	2925 [O2]
	387A	ARG	2.11	2.97	139.97	1615[Ng+]	2925 [O2]
30ES	320A	LYS	1.78	2.75	157.80	963 [Nam]	2939 [O2]
	331A	ASN	2.15	2.89	127.93	1086[Nam]	2936 [O-]
	467A	ASP	1.97	2.95	175.38	2947 [O3]	2348 [O2]

3. Hydrophobic Interactions of Lead Compounds for HK2

Name	Residue	Amino Acid	Distance	Ligand Atom	Protein Atom
11MS	67A	PHE	3.35	8142	500
	68A	VAL	3.55	8154	511
	162A	LYS	3.47	8149	1344
	163A	LEU	3.40	8152	1359
	456A	VAL	3.64	8153	4106
	459A	VAL	3.80	8156	4129
	459A	VAL	3.71	8155	4130
22MS	463A	LEU	3.88	8153	4175
	463A	LEU	3.37	8141	4176
	463A	LEU	3.33	8154	4173
25ES	68A	VAL	3.73	8132	511
	455A	MET	3.94	8141	4096
	459A	VAL	3.22	8135	4129
	459A	VAL	3.42	8131	4130
	462A	ARG	3.74	8154	4157
	463A	LEU	3.83	8143	4175
	463A	LEU	3.23	8154	4176
26ES	463A	LEU	3.56	8143	4176
	814A	ASP	3.87	8147	7248
26MS	67A	PHE	3.40	8139	500
	67A	PHE	3.80	8135	498
	162A	LYS	3.23	8132	1344
	163A	LEU	3.52	8136	1358
	463A	LEU	3.99	8146	4176
	463A	LEU	3.05	8136	4175
	817A	ILE	3.48	8149	7277
28MS	67A	PHE	2.85	8152	502
	163A	LEU	3.32	8145	1359
	248A	VAL	4.00	8155	2155
	456A	VAL	3.67	8146	4106
	459A	VAL	3.92	8145	4128
30ES	67A	PHE	3.63	8150	500
	69A	ARG	3.51	8143	518
	459A	VAL	3.01	8139	4130
	463A	LEU	3.31	8147	4175

4. Hydrophobic Interactions of Lead Compounds for GLS1

Name	Residue	Amino Acid	Distance	Ligand Atom	Protein Atom
11MS	322A	PHE	3.65	2943	992
	330A	HIS	3.00	2931	1070
	334A	VAL	2.94	2931	1111
	335A	ASN	3.87	2942	1118
	336A	ALA	3.08	2943	1129
	391A	ILE	3.01	2942	1658
22MS	322A	PHE	4.00	2947	992
	334A	VAL	3.49	2934	1111
	335A	ASN	3.82	2945	1118
	336A	ALA	3.19	2947	1129
	391A	ILE	3.49	2948	1658
25ES	334A	VAL	3.57	2935	1110
	334A	VAL	3.16	2925	1111
	466A	TYR	3.40	2930	2334
26ES	334A	VAL	3.03	2937	1111
26MS	330A	HIS	3.26	2928	1070
	334A	VAL	3.18	2931	1111
28MS	322A	PHE	3.97	2929	990
	330A	HIS	3.43	2950	1070
	334A	VAL	3.23	2950	1070
	336A	ALA	3.36	2929	1129
	466A	TYR	3.52	2943	2334
30ES	320A	LYS	3.42	2946	970
	334A	VAL	3.52	2942	1112

5. Pi Cation Interactions of Lead Compounds for HK2

Name	Residue	Amino Acid	Distance	Ligand Group	Ligand Atoms
22MS	69A	ARG	4.06	Aromatic	8134, 8135, 8136, 8137, 8138
26ES	462A	ARG	3.89	Aromatic	8151, 8152, 8153, 8154, 8155
30ES	69A	ARG	4.41	Aromatic	8146, 8147, 8148, 8149, 8150, 8152

6. Pi Cation Interactions of Lead Compounds for GLS1

Name	Residue	Amino Acid	Distance	Ligand Group	Ligand Atoms
28MS	320A	LYS	3.77	Aromatic	2944, 2938, 2939, 2940, 2941, 2943
	330A	HIS	4.18	Aromatic	2945, 2946, 2947, 2949, 2950, 2951

7. Salt Bridge Interactions of Lead Compounds for HK2

Name	Residue	Amino Acid	Distance	Ligand Group	Ligand Atoms
26MS	467A	HIS	5.07	Carboxylate	8142, 8150
	470A	ARG	4.09	Carboxylate	8142, 8150

8. Salt Bridge Interactions of Lead Compounds for GLS1

Name	Residue	Amino Acid	Distance	Ligand Group	Ligand Atoms
26ES	320A	LYS	4.67		2928, 2926
26MS	320A	LYS	3.75	Carboxylate	2944, 2936
	387A	ARG	5.08	Carboxylate	2944, 2936

9. Halogen Interactions of Lead Compounds for GLS1

Name	Residue	Amino Acid	Distance	Donor Angle	Acceptor Angle	Donor Atom	Acceptor Atom
28MS	319A	ASN	3.85	166.45	122.16	2948 [Cl]	959 [O2]
	508A	MET	3.64	146.96	138.99	2942 [Cl]	2696 [O2]
